# Exploring the genetic correlations of antisocial behaviour and life history traits

**DOI:** 10.1192/bjo.2018.63

**Published:** 2018-11-05

**Authors:** Jorim J. Tielbeek, J.C. Barnes, Arne Popma, Tinca J.C. Polderman, James J. Lee, John R.B. Perry, Danielle Posthuma, Brian B. Boutwell

**Affiliations:** Postdoctoral Researcher, Department of Complex Trait Genomics, VU University Amsterdam, the Netherlands; Associate Professor, School of Criminal Justice, University of Cincinnati, USA; Professor, Institute for Criminal Law and Criminology, Leiden University, the Netherlands; Assistant Professor, Department of Complex Trait Genomics, VU University Amsterdam, the Netherlands; Assistant Professor, Department of Psychology, University of Minnesota, USA; Doctor, School of Clinical Medicine, University of Cambridge, UK; Professor, Department of Complex Trait Genomics, VU University Amsterdam, the Netherlands; Associate Professor of Criminology and Criminal Justice, Department of Epidemiology and Department of Family and Community Medicine, Saint Louis University, USA

**Keywords:** Genome-wide association study, linkage disequilibrium regression, antisocial behaviour

## Abstract

Prior evolutionary theory provided reason to suspect that measures of development and reproduction would be correlated with antisocial behaviours in human and non-human species. Behavioural genetics has revealed that most quantitative traits are heritable, suggesting that these phenotypic correlations may share genetic aetiologies. We use genome-wide association study data to estimate the genetic correlations between various measures of reproductive development (*N* = 52 776–318 863) and antisocial behaviour (*N* = 31 968). Our genetic correlation analyses demonstrate that alleles associated with higher reproductive output (number of children ever born, *r*_*g*_ = 0.50, *P* = 0.0065) were positively correlated with alleles associated with antisocial behaviour, whereas alleles associated with more delayed reproductive onset (age at first birth, *r*_*g*_ = −0.64, *P* = 0.0008) were negatively associated with alleles linked to antisocial behaviour. Ultimately, these findings coalesce with evolutionary theories suggesting that increased antisocial behaviours may partly represent a faster life history approach, which may be significantly calibrated by genes.

**Declaration of interest:**

None.

A moderate proportion of the variance in antisocial phenotypes is accounted for by genetic variation.^1^ Phenotypic indicators of externalising traits, moreover, are correlated with other forms of psychopathological behaviour.^2^ These findings suggest a general vulnerability underlying the spectrum of externalising disorders, one underpinned by pleiotropic genetic influences. With the proliferation of population-based samples containing measured genes, researchers have investigated possible molecular correlates of antisocial behaviours. Although some promising results emerged, so too did to the recognition of limitations.^3^^,^^4^ Chief among them included the use of underpowered designs, lack of reproducibility and an absence of correction for multiple testing bias.^4^

As a result, studies utilising genome-wide techniques in large samples have become the preferred approach to unravelling the genetic architectures of complex traits (which have previously been shown to be moderately heritable).^4^^–^^10^ Recent genome-wide association studies (GWAS) of antisocial phenotypes revealed a number of trait-relevant alleles that were nearly genome-wide significant.^5^^,^^10^ GWAS evidence has also revealed associations between a number of single nucleotide polymorphisms (SNPs) and phenotypes known to correlate with antisocial behaviours.^6^ In particular, various indicators of reproductive behaviour, such as age at menarche, age at first sexual contact and number of sexual partners, have all been examined using genetically sensitive data, with results identifying a number of genome-wide significant SNPs.^6^

What remains in need of further testing is whether the genetic underpinnings of antisocial behaviour covary with the genetic underpinnings of reproductive indicators. For this topic, in particular, prior theoretical work has suggested that some forms of antisocial behaviour may represent natural variation in broad reproductive strategies – captured broadly in the area of life history evolution – that have been shaped by natural selection over the course of human evolution.^7^ This line of reasoning makes the explicit prediction that reproductive and antisocial behaviour should be correlated at a phenotypic level, and perhaps at a genetic level, such that alleles associated with antisocial behaviour are also associated with higher reproductive output and more rapid physical maturation. Indeed, in an earlier GWAS study, we examined the genetic covariation of antisocial behaviour with a range of cognitive, psychiatric and reproductive outcomes, utilising overlapping data, and reported some suggestive genetic correlations with various reproductive traits.^5^

We combined GWAS data from the Broad Antisocial Behavior Consortium (BroadABC) with data from the Early Genetics and Lifecourse Epidemiology (EAGLE) Consortium.^10^ In total, we meta-analysed genotypic and phenotypic data from 31 968 individuals across 13 unique samples, making it the largest collective sample available to estimate the effects of genome-wide genetic variation on antisocial behaviour. In addition, summary statistics for seven relevant reproductive and longevity traits were obtained from large existing GWAS data-sets. This study explores the genome-wide genetic correlation between reproductive traits and antisocial behaviour. By examining the extent to which alleles associated with antisocial behaviour are related to alleles underpinning variation in reproductive outcomes, this study could provide a better understanding of why these traits tend to correlate at the phenotypic level.^7^

## Method

In this study, we used (cross-trait) linkage disequilibrium score regression to estimate the SNP-based heritability of antisocial behaviour and reproductive traits and the genetic correlation between the traits explained by all SNPs.^8^ Linkage disequilibrium score regression disentangles the contribution of true polygenic signal and bias due to population stratification to the inflated test statistics in GWAS, and optionally calculates a genetic correlation (*r*_*g*_) between traits.^8^ An important condition is that the per-SNP heritability near a given SNP must not be confounded with the extent of that SNP's linkage disequilibrium with neighbouring SNPs. This condition is likely to be violated in the case of a phenotype related to fitness producing a downwardly biased, SNP-based heritability estimate.^8^ However, it is still possible for the genetic correlation between two traits to be estimated accurately given the bias in the numerator (the genetic covariance) cancelling the bias in the denominator (the square root of the product of two heritability estimates).

Here, we utilised linkage disequilibrium score regression to estimate the genetic correlation of antisocial behaviour with reproductive traits based on the summary statistics from the largest GWAS meta-analyses available. For reproductive traits, summary statistics were derived from the centralised database LD Hub.^9^ Moreover, we used PLINK to ‘clump’ the SNPs, utilising the 1000 Genomes V3 reference panel for Europeans, with 0.1 as the linkage disequilibrium *r*^2^ threshold, and 500 KB as physical distance threshold. Then, we examined whether the signs of the regression coefficients of the SNPs for antisocial behaviour and age at first birth (yielding the highest *r*_*g*_) were, more often than expected by chance, in the same direction. We tested this by a binomial test to verify whether the proportion of SNPs (yielding *P*-values <0.05) with concordant sign was higher or lower than expected by chance (0.5).

For antisocial behaviour, we performed a GWAS meta-analysis to obtain a large GWAS sample. We combined summary data from the publicly available EAGLE consortium (*N* = 18 988^10^) with those from nonoverlapping samples of the BroadABC (QIMR, TEDS, COGA and Yale-Penn; *N* = 12 980), totalling 31 968 participants. The genetic correlation between these two meta-analysed data-sets was 0.38 (s.e. 0.48). To maximise sample size, we included studies with a broad range of antisocial measures, including both aggressive and nonaggressive domains of antisocial behaviour, and utilising study-specific scales in different age groups.^5^^,^^10^^,^^11^ The meta-analysis was run using a fixed-effects model with *z*-scores weighted by sample size, as implemented in the software METAL.^12^

## Results

First, we calculated the SNP-based heritability estimates of antisocial behaviour and the life history traits, after which the genetic correlations between the traits were computed. The estimated heritability for antisocial behaviour utilising linkage disequilibrium score regression was low at 2.9% (s.e. 1.5%). Substantially higher estimates of the SNP-based heritability of antisocial behaviour have been reported elsewhere, using genome-wide complex traits analyses (GCTA) tools. We identified four cohorts (including QIMR, ALSPAC and GENR samples) with antisocial behaviour measures that previously performed GCTA, resulting in a sample-size weighted *h*^2^ of 35% (Table 1). These GCTA estimates are based on more homogeneous individual cohorts yielding smaller samples, which might explain the discrepancy in *h*^2^. The SNP heritabilities based on linkage disequilibrium score regression were 5.9% (s.e. 2.7%) and 5.2% (s.e. 2.7%), respectively, for the EAGLE and BroadABC consortia. Another possibility is that linkage disequilibrium score regression is more sensitive to causal SNPs being in low linkage disequilibrium with their neighbours.^12^

**Table 1:** Previously reported genome-wide complex trait analysis estimates on antisocial behaviour

Cohort, trait	*N*	SNP *h*^2^ ^c^	s.e.	*P-*value
QIMR^a^ (adult antisocial behaviour)	4816	0.55	0.41	0.07
ALSPAC^b^ (middle childhood/early adolescence)	5299	0.08	0.06	0.10
GENR^b^ (6-year)	2101	0.54	0.19	0.002
NTR^b^ (3-year)	908	0.46	0.35	0.09

a. Estimates included above were derived from studies by Tielbeek *et al* (2012).^11^

b. Estimates included above were derived from studies by Pappa *et al* (2016).^10^

c. SNP *h*^2^ is the estimation of narrow-sense heritability contributed by common SNPs. SNP, single nucleotide polymorphism; s.e., standard error

**Fig. 1:**
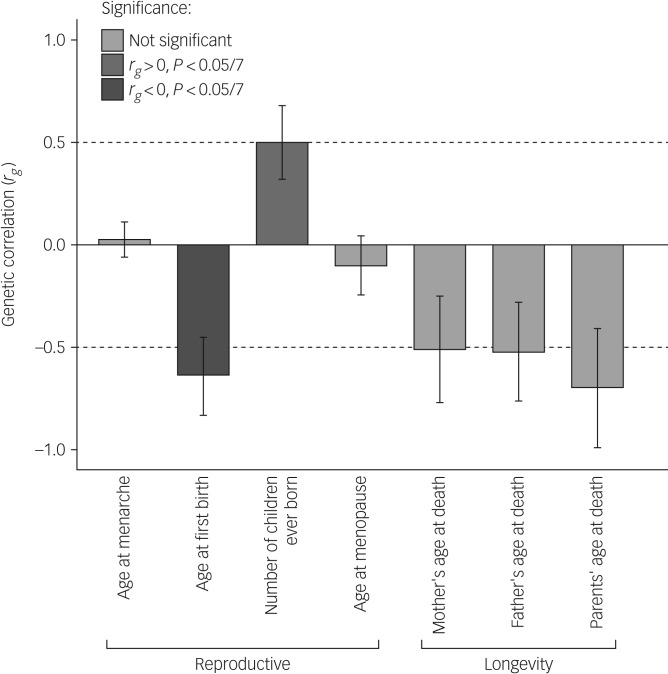
Genetic correlations between antisocial behaviour and life history variables.

Our main analysis (Table 2, Fig. 1) revealed significant (α < 0.0071) genetic correlations of antisocial behaviour with age at first birth (*r*_*g*_ = −0.64, *P* = 0.0008) and number of children ever born (*r*_*g*_ = 0.50, *P* = 0.0065), as well as suggestive associations with parents', mothers' and fathers' age at death, but not with age at menarche or age at menopause (r_g_ = 0.026, *P* = 0.76 and *r*_*g*_ = −0.10, *P* = 0.48, respectively). Moreover, sign tests revealed fewer SNPs yielding a *P*-value < 0.05 with the same direction of effect than expected by chance (proportion 0.47, *P* < 0.001) for antisocial behaviour and age at first birth.

**Table 2:** Genetic correlations across life history speed indicators (four reproductive traits and three longevity traits) and broad antisocial behaviour

Phenotype	Sample size	SNP *h*^2^	*r*_*g*_ (s.e.)	Bivariate intercept	*P-*value
Age at menarche	182.416	0.21	0.026 (0.085)	−0.0083	0.76
Age at first birth	222.037	0.063	−0.64 (0.19)	−0.0003	0.0008**
Number of children ever born	318.863	0.025	0.50 (0.18)	−0.007	0.0065**
Age at menopause	69.360	0.13	−0.10 (0.144)	−0.0016	0.48
Mother's age at death	52.776	0.040	−0.51 (0.26)	−0.0017	0.045*
Father's age at death	63.775	0.042	−0.52 (0.24)	−0.0036	0.026*
Parents’ age at death	45.627	0.032	−0.70 (0.29)	0.0093	0.015*

Genome-wide association study summary statistics from our combined analyses were used to calculate the *r*_*g*_ values with other traits. Single nucleotide polymorphism (SNP) *h*^2^ is the estimation of narrow-sense heritability contributed by common SNPs. *r*_*g*_ is the genetic correlation and is calculated with the LDSC software package, using pre-calculated linkage disequilibrium scores from Bulik-Sullivan *et al*.^8^

* Nominally significant (*P* < 0.05), ** Significant at the multiple-testing corrected *P*-value (0.0071).

## Discussion

This study expands on prior work,^5^ showing preliminary evidence of a genetic correlation between reproductively relevant traits and antisocial behaviour. Our genetic correlation analyses demonstrate that alleles associated with higher reproductive output (i.e. faster life history styles) were positively correlated with alleles associated with antisocial behaviour, whereas alleles associated with giving birth later in life were negatively associated with alleles linked to antisocial behaviour. It is important to acknowledge that this study is preliminary and not without limitations. Our correlations were somewhat higher than expected (ranging from 0.5 to 0.7). We hypothesise that with increasing samples and more homogeneous phenotypic measures, these estimates will be more in the range of 0.3–0.5, as reported by previous studies.^5^

Our genetic correlation analyses are based on, and limited by, relatively low SNP-based heritability estimates of antisocial behaviour (2.9%), yielding a heritability *z*-score of 1.93. The low SNP-based *h*^2^ estimate of antisocial behaviour may be because of heterogeneity in measurement by meta-analysing multiple cohorts or the tendency of the causal SNPs to be in low linkage disequilibrium with their neighbours.^13^ Finally, although somewhat elevated, the magnitude of the genetic correlations observed herein is similar in magnitude compared with other related phenotypes, utilising alternative samples.^5^^,^^14^ These results, although promising and guided by prior theory,^7^ should be viewed only as preliminary. As sample sizes continue to grow and a more diverse range of phenotypes become available for testing, additional insight should emerge regarding the molecular underpinnings for antisocial behaviour and human reproductive strategy.
